# Supplementary material for: Expression Dynamics of lpa1 Gene and Accumulation Pattern of Phytate in Maize Genotypes Possessing opaque2 and crtRB1 Genes at Different Stages of Kernel Development
Source: Plants (Basel). 2023 Apr 24;12(9):1745. doi: 10.3390/plants12091745 (PMC10180721; doi:10.3390/plants12091745)
Supplement: Supplementary file 1 [file plants-12-01745-s001.zip › plants-2254451-supplementary.pdf]

Table S1. Details of primers used for quantitative real-time PCR analysis

Gene	Gene bank accession	Primer sequences (5'→3')	Amplicon size (bp)
lpa1-1	EF586878	F-CTGTGCTTGGTTGGTGTCAT	117
		R-CTCCTCCTCTGCCTCTCTGC	
crtRB1	GQ889716	F: CATCTCCCTCCTCGCCTAC	91
		R: CATGCCGAACAGCGTAATC	
o2	X15544	F: GCCTCCAGTGTTGTGACCTC	103
		R: CGTTCATCAGCTTGTTCTGC	
Adh1	NC_050096.1	F: TTCAATCAATGGGAAGCCTA	135
		R: TTATCAAGGGGAGCCTGAG	

Table S2. Concentration of kernel phytic acid (PA) and inorganic phosphorous (iP) among the genotypes

Genotypes	*PA (mg/ g)*			*iP (mg/ g)*			*TP (mg/ g)*			*PA/TP (mg/ g)*		
	15 DAP	30 DAP	45 DAP	15 DAP	30 DAP	45 DAP	15 DAP	30 DAP	45 DAP	15 DAP	30 DAP	45 DAP
PMI-PV5	2.10	2.40	2.77	0.77	0.49	0.25	2.87	2.89	3.02	0.73	0.83	0.92
PMI-PV5-*lpa1*-A	1.16	1.45	1.87	1.06	1.02	0.99	2.22	2.48	2.86	0.52	0.59	0.65
PMI-PV5-*lpa1*-B	1.15	1.46	1.84	1.01	0.96	0.95	2.16	2.42	2.79	0.53	0.61	0.66
PMI-PV6	2.28	2.53	2.84	0.82	0.46	0.27	3.10	2.99	3.11	0.74	0.85	0.91
PMI-PV6-*lpa1*-A	1.10	1.37	1.69	1.09	1.03	1.02	2.19	2.40	2.72	0.50	0.57	0.62
PMI-PV6-*lpa1*-B	1.13	1.41	1.72	1.12	1.07	1.03	2.25	2.49	2.76	0.50	0.57	0.63
PMI-PV7	2.22	2.43	2.76	0.71	0.48	0.26	2.93	2.91	3.02	0.76	0.84	0.91
PMI-PV7-*lpa1*-A	1.08	1.42	1.77	0.95	0.91	0.92	2.03	2.33	2.69	0.53	0.61	0.66
PMI-PV7-*lpa1*-B	1.13	1.44	1.74	0.96	0.94	0.95	2.09	2.38	2.70	0.54	0.61	0.65
PMI-PV8	2.28	2.48	2.73	0.71	0.46	0.30	2.99	2.94	3.03	0.76	0.85	0.90
PMI-PV8-*lpa1*-A	1.18	1.43	1.75	1.03	0.97	0.96	2.21	2.40	2.71	0.53	0.60	0.65
PMI-PV8-*lpa1*-B	1.22	1.41	1.78	1.02	0.96	1.00	2.23	2.38	2.78	0.55	0.60	0.64
HKI1105	2.16	2.46	2.74	0.90	0.48	0.22	3.05	2.94	2.96	0.71	0.84	0.93
CD at 5%	0.06	0.07	0.07	0.06	0.05	0.06	0.10	0.10	0.11	0.02	0.01	0.01
SE	0.02	0.02	0.03	0.02	0.02	0.02	0.03	0.03	0.04	0.01	0.01	0.00

DAP: Days after pollination

Table S3. Concentration of kernel lysine, tryptophan and provitamin-A contents among the genotypes

Genotypes	*Lysine (% in protein)*			*Tryptophan (% in protein)*			*proA (µg/g)*		
	**15 DAP**	**30 DAP**	**45 DAP**	**15 DAP**	**30 DAP**	**45 DAP**	**15 DAP**	**30 DAP**	**45 DAP**
PMI-PV5	0.431	0.356	0.325	0.160	0.105	0.093	15.77	12.11	10.85
PMI-PV5-*lpa1*-A	0.451	0.369	0.334	0.150	0.101	0.089	16.26	12.37	10.93
PMI-PV5-*lpa1*-B	0.436	0.363	0.329	0.154	0.103	0.092	16.05	12.40	10.98
PMI-PV6	0.562	0.440	0.388	0.127	0.081	0.073	13.92	10.79	9.37
PMI-PV6-*lpa1*-A	0.564	0.445	0.398	0.132	0.088	0.077	14.01	10.62	9.28
PMI-PV6-*lpa1*-B	0.550	0.429	0.384	0.138	0.090	0.081	14.16	10.89	9.40
PMI-PV7	0.525	0.407	0.346	0.147	0.101	0.090	16.12	12.30	10.69
PMI-PV7-*lpa1*-A	0.486	0.385	0.334	0.161	0.108	0.094	16.87	12.51	10.72
PMI-PV7-*lpa1*-B	0.511	0.403	0.345	0.156	0.107	0.095	16.43	12.36	10.77
PMI-PV8	0.536	0.407	0.364	0.144	0.095	0.083	16.41	13.12	11.75
PMI-PV8-*lpa1*-A	0.547	0.419	0.373	0.139	0.092	0.081	16.84	13.30	11.82
PMI-PV8-*lpa1*-B	0.538	0.405	0.368	0.149	0.097	0.085	17.26	13.53	11.94
HKI1105	0.232	0.180	0.149	0.066	0.052	0.035	5.08	2.48	1.61
CD at 5%	0.0088	0.0081	0.0088	0.0086	0.0045	0.0064	0.14	0.18	0.09
SE	0.003	0.0028	0.003	0.0029	0.0015	0.0022	0.05	0.06	0.03

DAP: Days after pollination

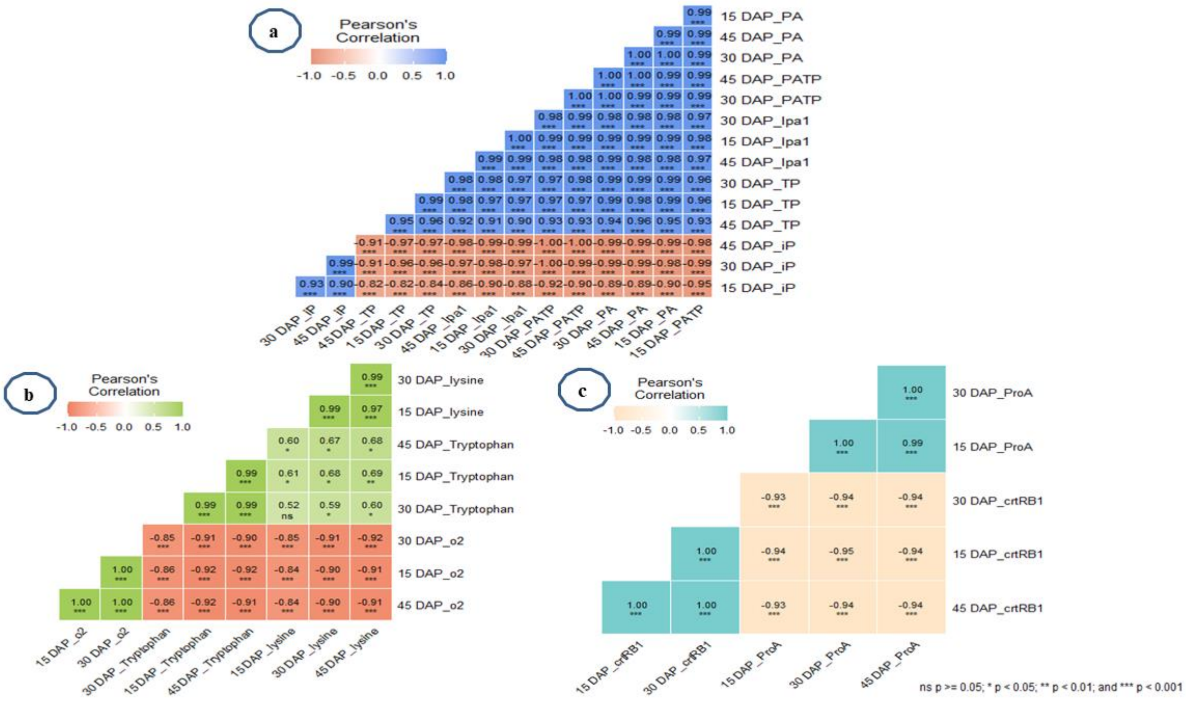

Figure S1: Correlation between gene expression and nutrient accumulation at 15, 30 and 45 days after pollination (DAP). (a) *lpa1-1* with PA, iP, TP and PATP; (b) *o2* with lysine and tryptophan; (c) *crtRB1* with provitamin-A. PA: phytic acid; iP: inorganic phosphorous; TP: total phosphorous; PATP: PA/TP; ProA: provitamin-A.
